# Dryland Soil Hydrological Processes and Their Impacts on the Nitrogen Balance in a Soil-Maize System of a Freeze-Thawing Agricultural Area

**DOI:** 10.1371/journal.pone.0101282

**Published:** 2014-07-07

**Authors:** Wei Ouyang, Siyang Chen, Guanqing Cai, Fanghua Hao

**Affiliations:** 1 School of Environment, State Key Laboratory of Water Environment Simulation, Beijing Normal University, Beijing, China; 2 Marine Monitoring and Forecasting Center of Zhejiang, Hangzhou, China; Zhejiang University, China

## Abstract

Understanding the fates of soil hydrological processes and nitrogen (N) is essential for optimizing the water and N in a dryland crop system with the goal of obtaining a maximum yield. Few investigations have addressed the dynamics of dryland N and its association with the soil hydrological process in a freeze-thawing agricultural area. With the daily monitoring of soil water content and acquisition rates at 15, 30, 60 and 90 cm depths, the soil hydrological process with the influence of rainfall was identified. The temporal-vertical soil water storage analysis indicated the local *albic* soil texture provided a stable soil water condition for maize growth with the rainfall as the only water source. Soil storage water averages at 0–20, 20–40 and 40–60 cm were observed to be 490.2, 593.8, and 358 m^3^ ha^−1^, respectively, during the growing season. The evapo-transpiration (ET), rainfall, and water loss analysis demonstrated that these factors increased in same temporal pattern and provided necessary water conditions for maize growth in a short period. The dry weight and N concentration of maize organs (root, leaf, stem, tassel, and grain) demonstrated the N accumulation increased to a peak in the maturity period and that grain had the most N. The maximum N accumulative rate reached about 500 mg m^−2^d^−1^ in leaves and grain. Over the entire growing season, the soil nitrate N decreased by amounts ranging from 48.9 kg N ha^−1^ to 65.3 kg N ha^−1^ over the 90 cm profile and the loss of ammonia-N ranged from 9.79 to 12.69 kg N ha^−1^. With soil water loss and N balance calculation, the N usage efficiency (*NUE*) over the 0–90 cm soil profile was 43%. The soil hydrological process due to special *soil* texture and the temporal features of rainfall determined the maize growth in the freeze-thawing agricultural area.

## Introduction

During the agricultural tillage management, the maximum crop production and minimum diffuse nitrogen (N) loading are the priority issues that need to be considered at the same time [1] [2]. It is essential to understand the fates of soil water (SW) and N in agricultural systems in order to attain higher crop yields and N usage efficiency [3]. In developing countries, there is much political and commercial pressure for controlling the N application with the sacrifice of the crop harvest [4]. The dryland agriculture in the Sanjian Plain, Northeast China, is a key food base and the most water limited agricultural zone in China [5]. In this freeze-thawing area, the soil N and water efficiency are the keys for dryland tillage sustainability due to the short growing season. However, there are few reports about the dryland soil hydrological process and N use efficiency in freeze-thawing agricultural areas [6].

The soil nutrient N is a major limiting factor for crop growth and production, which is directly related to the N fertilisation and soil N background level [7]. The soil microbial community, which affects the soil N cycle [8], is less active in this freeze-thawing area. The application of chemical N is a basic agricultural practice in worldwide, which causes excessive discharge of N to the aquatic environment [9]. The European Union agricultural landscape contributes about 55% of the diffuse pollution for water eutrophication. This is a major emerging environmental issue in the developing counties [10]. The soil eco-hydrological process is an essential channel for N transport in the soil-crop system.

Plant organs have different allocation rates with N during growth, and the canopy green area has higher N absorption than the other organs during the jointing period [11]. The N accumulations in the upper leaves of the canopy also follow the eco-hydrological pattern in farmland [12]. Some models have been developed to simulate the N absorption in different organs during crop growth with the impacts of the solar radiation, rainfall, temperature, soil hydrology and N concentration [13]. With the temporal pattern of N accumulation in crop organs, the N cycle and efficiency can also be analyzed. The diffuse N discharge from agricultural systems depends on diverse factors, including the climatic, soil properties and agronomic features [14] [15]. The tillage practice, soil hydrological process and N usage efficiency are the main potential factors to consider when the goal is to reduce the agricultural diffuse N pollution [16]. The dryland maize farmland in a freeze-thawing agricultural area presents different N discharge patterns due to the special hydrological process [17].

The soil hydrological process is the bridge for diffuse N loss from soil to water bodies [18]. The nitrate-N is the dominant type of diffuse N in the soil profile and it can move swiftly downstream flow with the rainfall through the soil pathways [19]. The soil hydrological process is also closely related to the soil texture, which is the medium for water movement and crop growth. In the freeze-thawing dryland, the soil has short-term, transitional phase and rainfall is the only water source for N movement with crop growth [20]. The soil pattern in the study area is meadow *albic* soil (*Albic Luvisols*) and has a 20–30 cm depth impermeable stratum. The *albic* soil provides a stable soil water (SW) condition in the crop root zone and interfaces with the soil hydrological process. With the consideration of high N efficiency, it is necessary to analyze the special pattern of soil hydrology and its impact on the N efficiency [21]. Evaluating the response of N loss to the combination of rainfall and the soil hydrological process can help to identify the interactions of water with N and reduce diffuse N pollution.

The general objective is to maximize the N efficiency in tillage practices. Typically, of about 20%–70% of N is lost from soil-crop systems and this loss can cause other environmental issues [22]. The quality of soil properties will decline under the combination of intensive application of N fertilizer and low N efficiency; this in turn, will also deliver some risk to water [23]. There are several methods to improve the N efficiency, including accurate fertilizer application and irrigation [24] [25]. The dryland soil hydrological process in a freeze-thawing agricultural area presents a special pattern and also affects the N efficiency [26]. This study aims (1) to identify the temporal-vertical dynamics of dryland SW within an *albic* soil layer; (2) to express the dry weight and N accumulation of maize organs in dryland of a freeze-thawing agricultural area; (3) to explore the variation of soil loss, potential N loss, and N use efficiency (*NUE*) in dryland during the growing season.

## Materials and Methods

### 2.1 Ethics statement

The field experiment was conducted in the farmland of Bawujiu Farm, which has been rented by Wang Dongli for 50 years. He is also the contact person for future permission. No specific permissions were required for these locations. The field studies did not involve endangered or protected species. This study did not involve vertebrate. The specific location of our study is 133° 50′–134° 33′ E, 47° 18′47°50′ N.

### 2.2 Study area description

**Figure 1:**
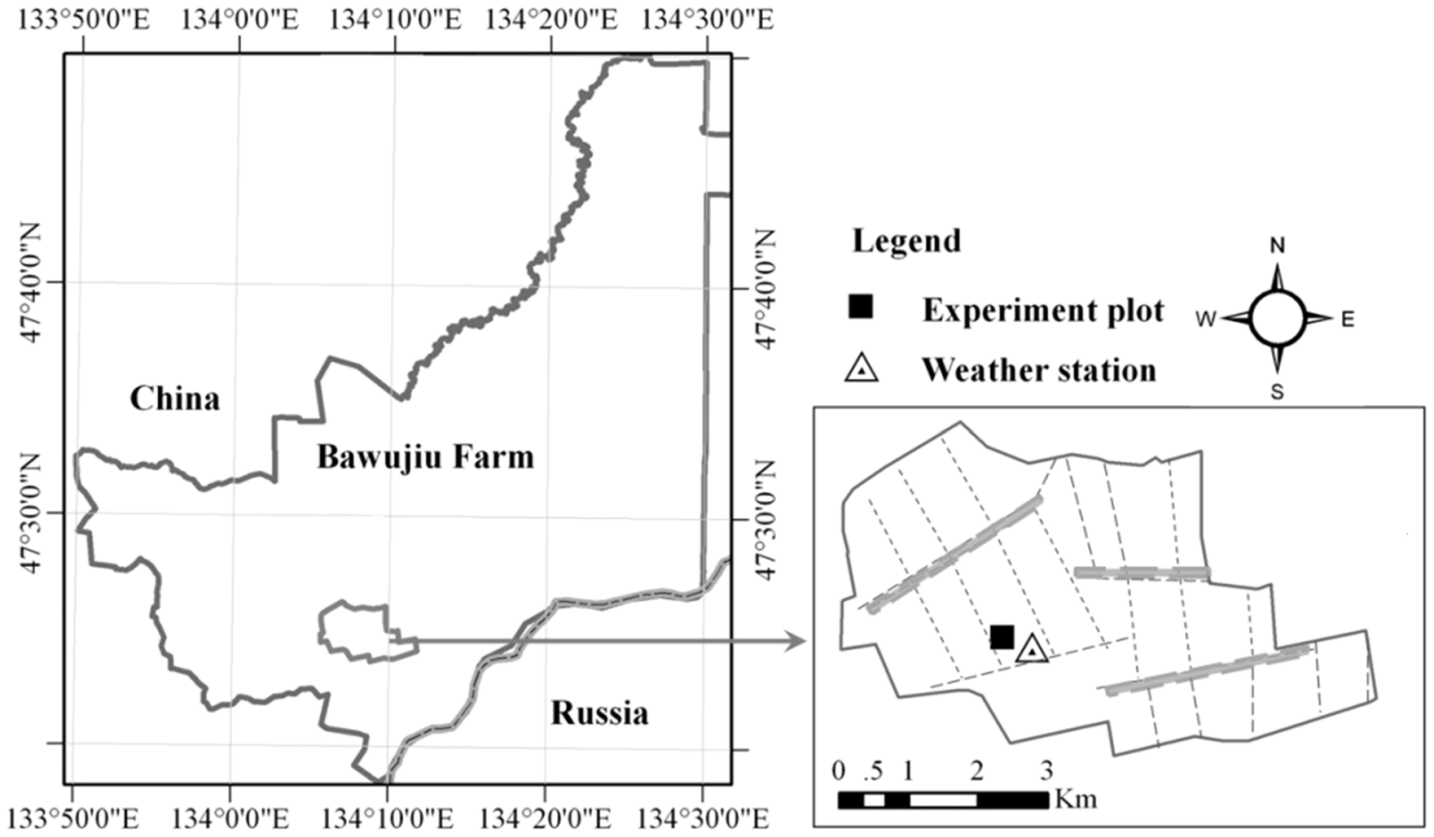
Location of the experimental dryland site in Northeast of China.

The case study area was conducted on a farm in the north-eastern part of China. The farm has a long history and on the east is adjoined to Russia (Fig. 1). The observation site is located in the southern part of the Farm and the elevation is 48 m. The only water source for dryland tillage is precipitation and maize is the dominant crop. This area the temperate continental monsoon climate has an average annual precipitation of 588 mm and an average yearly temperature of 2.94 °C [27]. From October to the following April, the temperature is below zero and, as a result, the soil freeze-thawing process affects the tillage process. The crop growing season is from May to October.

### 2.3 Field monitoring and soil sample collection

In order to identify soil physical-chemical properties, soil samples at four depths (0–15, 15–30, 30–60 and 60–90 cm) were collected in 2010. The samples from three points chosen at random in each layer were mixed into a composite soil sample and placed in plastic bags for lab measurements. The soil samples were collected before sowing and after harvest. Soil particle size distributions were measured by laser diffraction after the removal of organic residue (MasterSizer S, Malvern Instruments, Malvern, UK). The soil's total nitrogen (TN) and organic carbon (OC) concentrations were measured with a CHN Elemental Analyser (Euro VectorS.P.A EA3000, 136 Milan, Italy) [28]. The ammonia N in rainfall was analyzed with the aid of Nessler's reagent and the nitrate-N concentration was determined by the Ultraviolet Spectro Photometric Method. The soil's pH was determined with a pH meter (METTLER TOLEDO, Switzerland) after mixing the fresh soil with deionised water (1∶2.5, w/v) [24]. The soil N stock at four depths was calculated with the following equation,
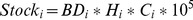
(1)where Stock*_i_* is the soil N storage at layer *i* (kg/m^2^), *BD_i_* is the soil bulk density at layer *i* (g/cm^3^), *H_i_* is the soil depth at layer *i* (cm), and *C_i_* is the soil N concentration at layer *i* (mg/kg).

For the soil hydrological process monitoring, the SW collector head (suction cup: Teflon and quartz; OD 21 mm×L 95 mm; porosity: 2 µm; conductivity: 3.31×10^−6^ mm sec^−1^, PRENART, Danmark) was set at 15, 30, 60 and 90 cm depths. It was covered with a mixed solution of quartz powder and stirred, which can cause a 0.05 MPa vacuum pressure and in direct contact with the soil. The collector tail was connected with PVC pipe and the water samples were collected in bottles by a portable manual vacuum pump. The soil water storage was calculated with soil volumetric water content at different depths. The mean soil water storage at each layer was defined as the average of SW in the upper and lower soil layers. The evapo-transpiration in dryland is calculated with the Bowen ratio and energy balance (BREB). The SW balance was calculated with the following equation [29],
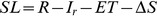
(2)where *SL* is the soil water loss (mm), *R* is the precipitation (mm), *ET* is the evapo-transpiration (mm), *I_r_* is the canopy interception (mm), and Δ*S* is the soil water storage change (mm).

The local meteorological characteristics were monitored with a ZENO station (Coastal, Seattle, WA, USA). Automated soil temperature sensors (Thermistor, Coastal, Seattle, WA, USA) and soil volumetric water content sensors (TDR type, Coastal, Seattle, WA, USA) were placed in each of the four different soil layers. The monitoring sensors were repeated at double at horizontal 0.5 m space.

### 2.4 Crop sample collection and measurement

The local dryland is intensively cultivated with the aid of mechanized equipment and the maize (*Zea mays L.*) planting density is 75,000 plants ha^−1^. The detailed maize planting date, harvest date, relevant tillage and fertilization practices are listed in Table 1. The maize samples in 2011 were collected monthly and the sample in 2010 were only collected in October due to the limited experimental conditions. The detailed process of sample collection and monitoring of crop features (height (CH), leaf area index (LAI) and root depth (RD)) are listed in a previously published paper [24]. The maize samples were taken to the lab immediately and the root, leaves, stems, tassels, and grains were separated. The samples were washed clean and then dried for 48 h at 65°C. The dry weight of organ samples was determined after fixing the temperature for 30 min at 105°C. The dried maize samples were crushed and sieved for crop organ N content (%) analysis (Vario EL, Elementar Co. Ltd., Germany). The N uptake amount by crop was calculated based on crop dry weight and plant N content (%).

**Table 1:** Dryland tillage practices and fertilization information.

Crop year	Planting date	Harvest date	Tillage prior to planting	Main fertilisation(kg ha^−1^)
				
2010	8/Jun.	9/Oct.	Tilling	525
2011	30/May.	5/Oct.		525
Detailed fertilisation data and amounts * (kg ha^−1^)				
2010	2011	N	P	K
8/Jun.	30/May.	71.25	58.65	27.23
27/Jun.	20/Jun.	34.50	13.80	10.02
5/Jul.	1/Jul.	29.45		

*Based on computation of the N, P, and potassium (K) elements.

### 2.5 Nitrogen supply and loss calculation

The N use efficiency (*NUE*) in a crop-soil system is defined as the ratio of N uptake (*N_c_*) by a crop to N supply (*N_s_*) in a system [30]. The *N_s_* was defined as the total available N to the crop, which included fertilizer N (*N_f_*), mineralized N (*N_m_*), initial mineralized N in soil (*N_min initial_*), N fixed by soil (*N_x_*) and N deposition (*N_d_*) from rainfall. The *N_s_* is calculated as:
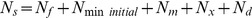
(3)


The field *N_m_* is calculated with the crop-soil N equation [31]:
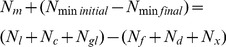
(4)where *N_m_* is the mineralized N, *N_min initial_* is the initial mineralized N concentration in soil, *N_min final_* is the final mineralized N concentration in soil, *N_l_* is the soil loss N, *N_gl_* is the N loss in gas. *N_c_* is the N crop absorption, *N_x_* is the N fixed by soil, and *N_d_* is the N deposition.

The mineralized N is calculated with the following equation,
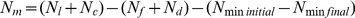
(5)


The potential soil water N loss (*N_l_*) is defined as the total loss of nitrate and ammonia, which was estimated with the N concentration in SW and the water volume in dryland [32]. Based on the SW loss and the N concentration in SW at different depths, the soil N loss by water was calculated.

### 2.6 Data analyses

Data analysis was performed with Sigma plot 10.0 and SPSS 16.0 software. The differences in dry weight and their percentages on crop organs (including root, leaf, stem, tassel, grain) in different growing stages were tested with ANOVA. The difference in the crop N uptake allocation proportion, the N accumulation in different organs, and the mineralized N concentration in different soil layers were statistically analyzed by ANOVA. The multiple comparisons were analyzed with the Duncan method.

## Results

### 3.1 Temporal-spatial distribution of soil water in dryland

**Figure 2:**
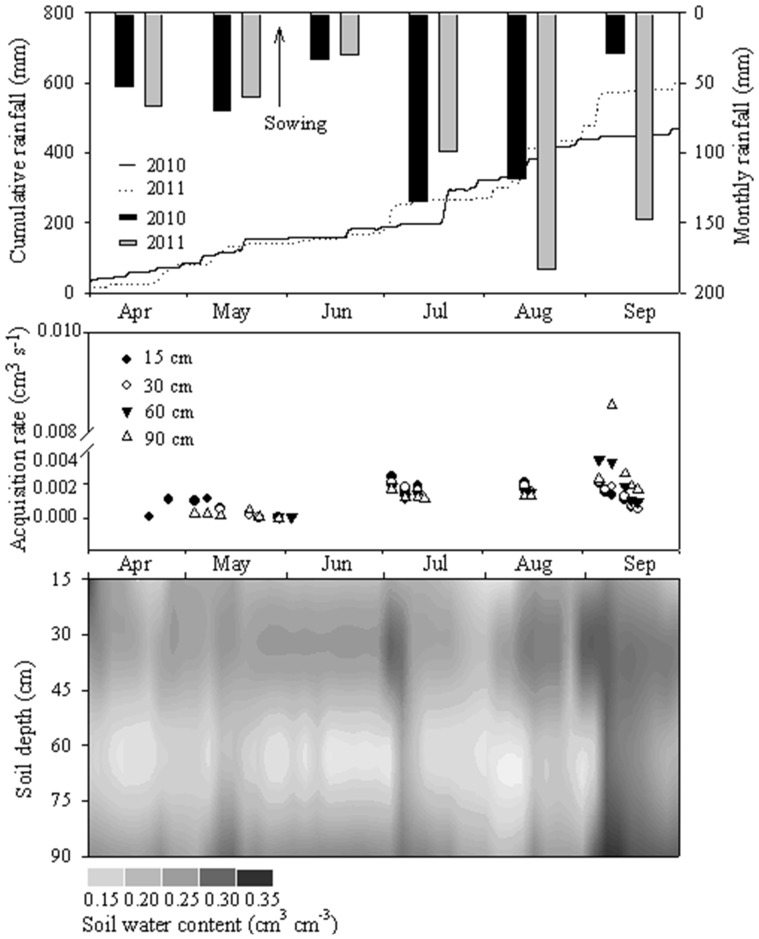
Daily precipitation in 2010 and 2011, and acquisition rate, and soil hydrological pattern of dryland in 2011.

With the soil-maize water monitoring system, the daily precipitation, SW acquisition rate, and SW content in the entire growing period was determined (Fig. 2). The monthly precipitation in the years 2010 and 2011 displayed a similar pattern, except for the lower value in September 2010. The precipitation occurred mainly between July and September, which was also the prime period for maize growth. With the light precipitation in April and May, the maize seed was sowed in moist soil. The precipitation in August and September of 2010 was 64 mm and 120 mm less than in the corresponding months of 2011. The cumulative precipitation from April to September in 2011 was 575 mm, which was 141 mm more than that in 2010. The precipitation was the only water source for the dryland in this freeze-thawing agricultural area, which directly impacted the SW acquisition rate. The SW could be collected only after the rainfall and the vertical difference in the SW acquisition rate was slight (around 0.002 cm^3^ s^−1^). In the entire monitoring period, the biggest acquisition rate occurred at the 90 cm depth after the rainfall in early September. The direct reason for it was that only smaller amount of the water can move deeply in the vertical direction after the absorption by roots.

The SW content increased in a stable manner during the growth period and also coincided with the accumulative precipitation. In all of June, the SW content at the 30 cm depth was the highest, the reason for this was that uptake of water in growing roots resulted in water accumulation in the rhizosphere. Due to the 73.6 mm rainfall on July 3, the SW content at the 30 cm depth increased by 20% to 0.30 cm^3^ cm^−3^. In the next two days, the SW content was transported in the vertical direction and it increased to 0.30 cm^3^ cm^−3^ in the deep layer. This vertical distribution also confirmed the retention role of roots in the early growing season. The surface SW content increased significantly after August due to the precipitation and then was transported to the deep soil layers. Later, the impact of the crop roots decreased and the soil texture was the main factor for SW transport in September.

In order to further analyse SW content fluctuation in the temporal-vertical dimensions, a statistical analysis was performed on the data from the soil layers during the growing season (30 May–30 September) (Table 2). The SW content averages at the 15 cm and 60 cm depths were very close with values of 0.245 and 0.230 cm^3^ cm^−3^, respectively. The mean SW content at the 30 cm and 90 cm depths were higher than at the other two layers with values of 0.272 cm^3^ cm^−3^ and 0.280 cm^3^ cm^−3^, respectively. The close relationship between the SW acquisition rate and the SW content that was shown in Fig. 2, was also confirmed by the statistical results. The SW acquisition rate had the same vertical pattern as the SW content and the higher values appeared at the 30 and 90 cm depths. Before maize seeding (May 30), the SW could be collected when the SW content reached about 0.25 cm^3^ cm^−3^. After maize seeding, the SW could be collected when it reached a content of over 0.30 cm^3^ cm^−3^. The SW acquisition rate was greatly and primarily influenced by rainfall.

**Table 2:** Statistical analysis of soil water content and acquisition rate at four depths during maize growing period.

Depth (cm)Index	Soil water content (cm^3^ cm^−3^)				Soil water acquisition rate (cm^3^ s^−1^)			
	15	30	60	90	15	30	60	90
Minimum	0.210	0.235	0.205	0.240	0.00008	0.00005	0.00009	0.00003
Maximum	0.308	0.333	0.306	0.342	0.00241	0.00208	0.00324	0.00856
Mean	0.245	0.272	0.230	0.280	0.00125	0.00114	0.00140	0.00167
Std. Error	0.018	0.020	0.024	0.024	0.00077	0.00078	0.00091	0.00220
Coefficient of variance	0.074	0.072	0.106	0.088	0.615	0.685	0.648	1.320

### 3.2 Temporal variation of soil water storage

**Figure 3:**
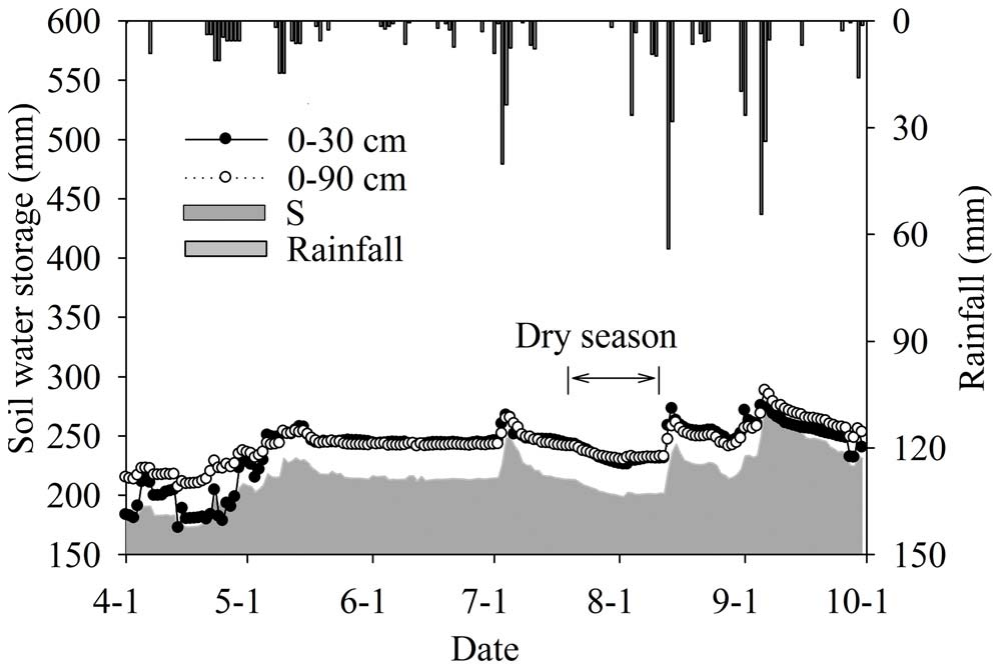
Temporal patterns of daily rainfall, soil water storage (SWS) and volumetric water content at 30 and 90 cm depths.

Based on the monitoring of the SW characteristics, the soil volumetric water content (SVW) and soil water storage (SWS) in the top 30 and 90 cm were calculated, respectively (Fig. 3). The SVW and SWS of the 0–90 cm and 0–30 cm depth showed similar temporal trends. In the vertical dimension, the SVW of the same two depths (0–30 cm and 0–90 cm) had similar trends in most periods, except in the first month which revealed a difference due to thawing. The thawing process from the surface to the deep layer and the tillage in preparation for sowing in the top soil decreased the SVW at 0–30 cm depth. There was almost no difference in the SVW between the two depths from mid-May to August. Before the crop harvest period in September, the deeper soil had a higher SVW content than the surface layer due to less root activity and strong precipitation. The SWS of the 0–30 cm depth had a seasonal variation and had a more direct relationship with precipitation events. Before the rainfall on 1 July, the SWS remained at a stable level from mid-May to the end of June. The SWS had a periodic decrease when no rainfall occurred in late July. The SWS reached its summit with the large rainfall in the later of August.

The SWS was calculated with the vertical variation in the SVW. The statistical characteristics of SWS and their proportions at each depth are listed in Table 3. The soil water storage averages at 0–20, 20–40 and 40–60 cm were 490.2, 593.8, and 358 m^3^ ha^−1^. The vertical differences indicated the middle level has a higher soil water content as a consequence of soil texture and crop root zone influences. Because precipitation was the only water source for dryland, the SWS at the three layers in the dry period was smaller than the average for the entire growing season, which also revealed the impact of maize ET on SWS. The SWS in the vertical dimension had the same trend in the entire period and the middle layer had the largest value (548.9 m^3^ ha^−1^). The differences in SWS between the two periods become greater when going from the surface to the deeper layer, an observation derived from the rainfall contribution to the SWS in deeper layer.

**Table 3:** Dryland soil water storage and its proportion at three depths.

Depth/cm	DurationItem	Entire growth season (30 May.–30 Sep.)	Dry season (20 Jul.–12 Aug.)
0–20	SWS/m^3^ ha^−1^	490.2±34.6	441.1±19.5
	Proportion/%	23.4±1.04	23.3±0.4
0–40	SWS/m^3^ ha^−1^	1084±62.9	990.0±55.9
	Proportion/%	51.8±2.6	52.0±0.76
0–60	SWS/m^3^ ha^−1^	1442±111	1303.0±70.6
	Proportion/%	68.8±1.6	68.5±0.55

**Figure 4:**
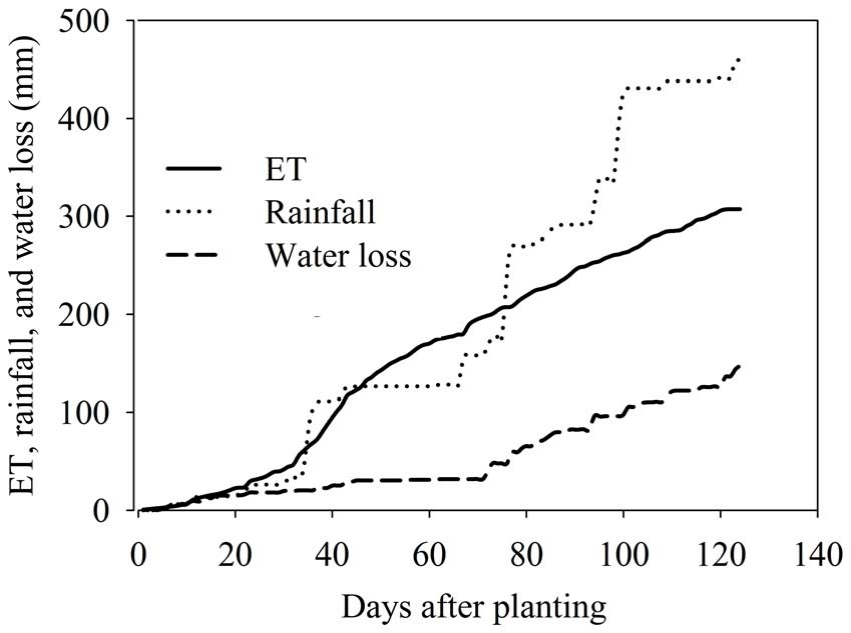
Cumulative evapo-transpiration (ET), rainfall, and water loss throughout maize growth period.

Based on daily monitoring data, the accumulative patterns of evapo-transpiration (ET), rainfall, and water loss in the growing season were compared (Fig. 4). The ET was at a low level in the first month of the growing period and then assumed a stable increasing rate beyond the 50th day after sowing. The accumulated SW loss was consistent with the fluctuation in the rainfall. The ET and rainfall stayed with the SW loss over the first 22 days. The accumulated rainfall amount increased by 79.8 mm; a large increase occurred on the 35th day when the ET and water loss increased slightly. The rainfall increased intensively after 80 days, which also caused an increase in the SW loss. The accumulated amounts of rainfall increased from 269.3 mm after 80 days to 440.9 mm after 120 days, and the SW loss increased from 47.9 mm after 75 days to 126.3 mm after 120 days. The temporal pattern demonstrated that the SW loss and ET of dryland were strongly correlated with the rainfall. The ET was also a maize growth feature and its similar pattern with rainfall also proved it was the dominant factor for crop growth.

### 3.3 Variation of crop biomass in the growing period

**Figure 5:**
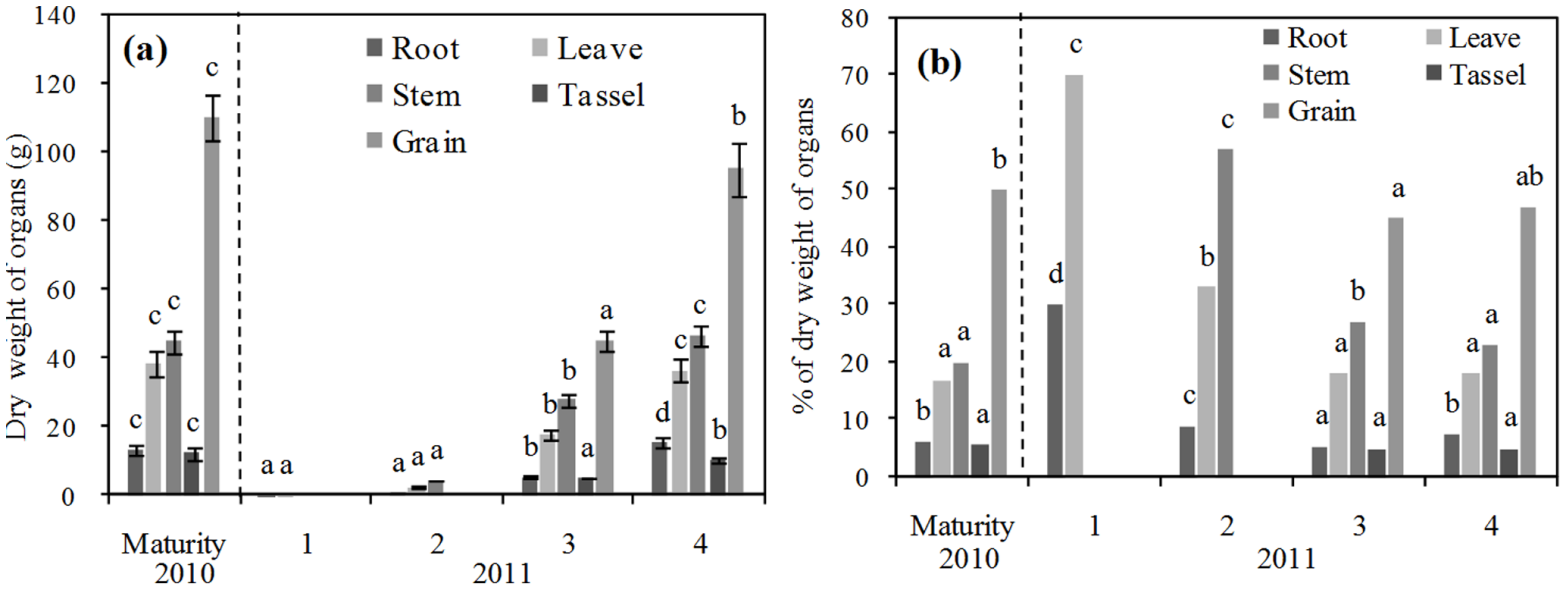
Dry weights (a) and percentage of maize organ weights (b) at different growth stages (Same letters in same organ between different growth stages indicate no significant difference. 1-Seedling, 2-Jointing, 3-Grain filling, 4-Maturity).

The dry weight of the different maize organs (root, leaf, stem, tassel, and grain) in growing stages were analyzed (Fig. 5). Also shown in the Fig. 5 are the percentages of the total dry weight comprised by each organ in each of four growing seasons. The dry organ weight varied greatly from the seedling stage to maturity, a development period which is relatively short in this freeze-thawing area. The temporal pattern of dry weight in 2011 revealed the changes that occurred over four growing sections. The dry weight of grains increased significantly in the grain filling and maturity sections. The dry weight percentage did not change as intensively as the weight, and it was also found that the grains comprised nearly half of the maize weight in the maturity period. With the N concentration in dried organs, the N absorption amount was quantified along with the organ dry weight. Due to logistical limitations, the crop organ sample in 2010 was only collected in the maturity period. The grain dry weights were larger in 2010 than in 2011. Comparing the dry weights of the organs in the maturity stage of the two years, the dry weights of the roots and tassel were significantly but their percentages differed little.

### 3.4 Nitrogen uptake by crop and accumulation

**Figure 6:**
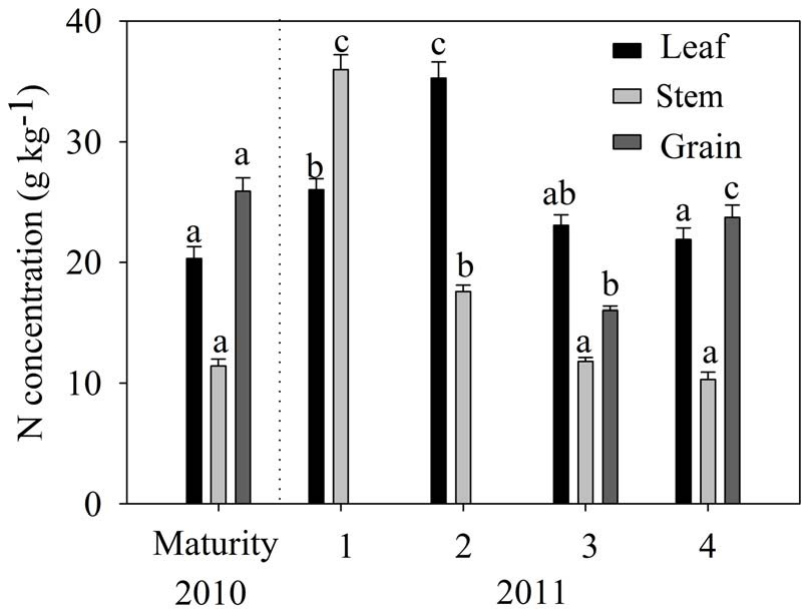
Seasonal dynamics of N concentration in maize organs (Same letters in same crop organs indicates no significant change between different growth stages. 1-Seedling, 2-Jointing, 3-Grain filling, 4-Maturity).

The temporal N dynamics of crop uptake was then analyzed with the samples obtained from the maize organs (Fig. 6). As the maize grew, the N uptake in leaf first increased and then decreased with the maximum occurring in the jointing period. From grain filling to maturity, N uptake by leaf diminished insignificantly with a relative flat trend compared with that from the jointing to grain filling stages. As maize grew N uptake in stem decreased significantly decrease from 1 to 2 with a probable transfer of much N to tassel. The difference of N uptake by stem in the grain filling and maturity stages was insignificant, but the N uptake by grain increased significantly (P<0.001). The N uptake by leaf decreased insignificantly, which indicated that a large amount of N was transferred to tassel in the early time period and then transferred from tassel to grain (N uptakes by tassel in grain filling and maturity were 8.12 g kg^−1^ and 3.57 g kg^−1^, respectively). There were insignificant differences in N uptakes of stem and leaf during the maturity stage in 2010 and 2011. The N uptake by grain in 2010 was 9.1% more than that in 2011.

The accumulated N in different organs gradually increased in the early stages and reached a summit in the maturity period. From the jointing to grain filling period, the N in stem increased about 4.23 times and the amount in leaf increased less than five times. The N amounts in crop organs were not only related to the N uptake efficiency, but they also depended on the organ dry weight. According to the analysis in the maturity period, grain accumulated most of N, which occurred in a short time section. The total N amount of maize organs in 2010 was 309.9 kg ha^−1^, which was larger than in 2011; the difference in grain was the dominant factor. The differences of N accumulation in leaf and stem during the maturity stages in the years 2010 and 2011 were insignificant.

**Figure 7:**
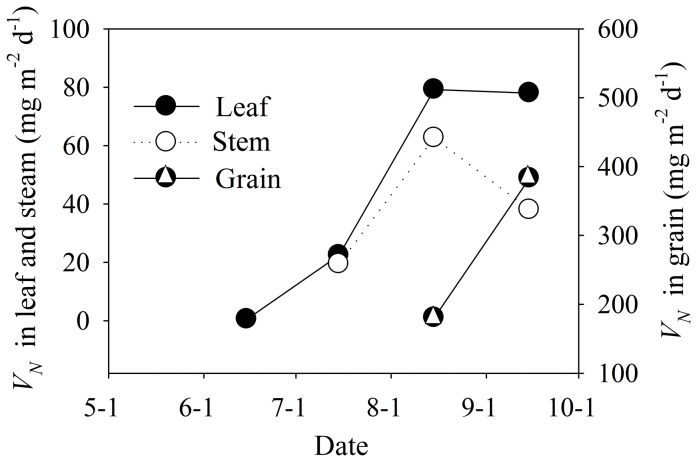
Seasonal dynamics of N accumulative rates (VN) in maize organs.

Seasonal dynamics of N accumulative rates in organs during the growth period were analyzed (Fig. 7). From June to August, the N accumulative rates of stem and leaf increased rapidly and then declined. When the stem and leaf accumulated less N, the absorption efficiency in grain increased to 383 mg m^−2^d^−1^. The leaf was the most effective organ in uptaking N and the accumulation rate was about 500 mg m^−2^d^−1^.

### 3.5 Variations of soil N stocks in the growing season

The N concentrations at four depths before sowing and after harvest were analyzed, which demonstrated the temporal-vertical soil N dynamics. The N (nitrate and ammonia) concentrations at different depths were first analyzed after the growing season (Table 5). Before the sowing, the ammonia-N stock at four depths ranged from 18.8 to 21.3 kg N ha^−1^ with no occurrence of a significant difference. After the harvest, the deeper layer experienced more loss than the surface layer. The middle two layers had close values and the 12.5 kg N ha^−1^ in top layer was the largest value. The nitrate-N concentration in soil was much larger than the ammonia-N. At the beginning of the growing season, the nitrate-N concentration was more than 121 kg ha^−1^ at all depths and had slight differences at 60–90 cm depth. The nitrate-N concentration decreased to 90 kg ha^−1^ after the harvest. The upper two layers had similar patterns and the concentrations ranged between 88–90 kg ha^−1^. There was no significant difference in the concentration in the deeper two layers and their concentrations ranged from 63.2 to 68.0 kg ha^−1^. The nitrate-N was more variable than ammonia-N and had a larger stock gap.

**Table 4:** Temporal pattern of N amount (kg ha^−1^) of maize organs during growing season*.

Year	Growth stage	Leaf	Stem	Grain
2010	Mature	58.6f	38.3ef	213de
2011	Seedling	0.14a		
	Jointing	6.85c	5.81a	
	Grain filling	30.6d	24.6d	54.1b
	Maturity	60.0f	36.0e	169c

*Same lowercase letter in one column indicats no significant difference (P<0.001); Same uppercase letter in one row indicates no significant difference (P<0.001).

**Table 5:** Mineral N stocks (kg ha^−1^) and variations at each depth of growing season.

Depth (cm)	Before sowing		After harvest		Stock gap	
	NO_3_^−^-N	NH_4_^+^-N	NO_3_^−^-N	NH_4_^+^-N	NO_3_^−^-N	NH_4_^+^-N
0–15	147d	21.3b	90.1d	12.5c	−48.9c	−8.64bc
15–30	144d	21.2b	88.1d	8.12b	−53.8cd	−11.2c
30–60	130cd	19.3b	68.0c	9.02b	−65.3e	−9.79c
60–90	121c	18.8b	63.2c	5.74a	−56.8d	−12.69c

Same letters in one column indicates no significant difference (P<0.001).

### 3.6 Nutrient loss and supply in soil-crop system

Based on field water monitoring and SW content in the 0–90 cm profile, the soil water loss (SL) and the leached N were calculated (Table 6). The rainfall and evapo-transpiration (ET) were calculated in the four stages of the maize growing season. The SL had a closer correlation with rainfall than with ET. The rainfall was nearly 100 mm in the jointing stage, but the SL was 11.5 mm due to the intensive ET. Moving from the grain filling stage to the maturity stage, the ET increased and more SL occurred under the higher rainfall event. The leached N was also calculated with the N concentration in soil water. The nitrate-N was the dominant N lost from the 90 cm soil profile, especially in the grain filling and maturity stages. The leached ammonia-N had similar temporal patterns and the largest amount was 0.18 kg ha^−1^.

**Table 6:** Amounts of soil water loss (SL) and N leached below maize root zone (90 cm).

Growth stage	Period	R	ET	SL	leached N (kg ha^−1^)	
		(mm)			NO_3_^−^-N	NH_4_^+^-N
Seedling	30 May to 29 Jun.	29.8	44.6	19.6	0.03	0.01
Jointing	30 Jun. to 25 Jul.	96.5	119.0	11.5	3.36	0.12
Grain filling	26 Jul. to 23 Aug.	165	67.3	48.6	5.53	0.18
Maturity	24 Aug. to 30 Sep.	168	76.6	67.5	6.26	0.10

Based on the N monitoring of rainfall and the survey of N fertilization, the N in rainfall and fertilizer was calculated. With the SWS and N concentration in SW in 0–90 cm profile, the *NUE* in the maize-soil system was calculated (Table 7). The N input from rainfall and fertilizer in the entire maize growing season was 16.5 and 120 kg ha^−1^, respectively. The soil N supply in the entire growth period was about 641 kg ha^−1^. With the nitrate and ammonia concentrations in SW and water volume, the N loss was estimated at 15.6 kg ha^−1^. Based on the N balance, it was calculated that the *NUE* was 43%. On the other hand, the excessive N from dryland was an important potential source for N discharge to the environment.

**Table 7:** The N balance and usage efficiency (*NUE*) in crop-soil system during the maize growth period.

Rainfall	Fertilizer	Initial soil N		Final soil N		N loss		Soil supply N	*NUE*
(kg N ha^−1^)		(kg N ha^−1^)		(kg N ha^−1^)		(kg N ha^−1^)		(kg N ha^−1^)	(kg kg^−1^)
		NO_3_^−^-N	NH_4_^+^-N	NO_3_^−^-N	NH_4_^+^-N	NO_3_^−^-N	NH_4_^+^-N		
16.5	120	542.9±29.76	80.6±4.29	309.4±18.4	35.4±4.37	15.2	0.41	641±14.4	0.43±0.03

## Discussion

### 4.1 The dryland soil hydrological process in a freeze-thawing area

Rainfall is the only water source in dryland and it has a direct impact on SW content and storage [33]. The field monitoring done in four stages as part of our study indicated that the temporal patterns of SW content and acquisition rate closely followed the rainfall amount (Fig. 2). The soil roughness in a cultivated area is increased by tillage, which enhances the vertical transport of SW [34]. The SL was 146.7 mm (32% of rainfall in the growing season). However, the vertical distribution of SW content and soil water storage demonstrate that the water has a short vertical movement range. The SW can be sampled only after the rainfall events, but the acquisition rates showed slight vertical differences. These two findings both demonstrated that the vertical movement of water is slow even with large volumes and the speed can affect the range.

Compared with the long growing periods in other dryland agricultural areas, the local maize growing season is short and has a higher efficiency in the use of water and N [35]. A proper SW condition in the root zone is essential for crop growth in the maize seeding and grain filling stages. The SWS can remain at a stable level (about 1000 m^3^ ha^−1^ of 40 cm profile) even in the dry season without precipitation (Fig. 3). All of these factors indicate that the *albic* soil texture can provide a positive water condition for crop growth [24]. The SW also controls surficial N movements in the dryland and provides a strong signal for crop absorption [36]. Crop transpiration will increase when the maize is near its maturity period, and the precipitation normally increases and meets this need during the same period (Fig. 4). The combined conditions of special soil texture and precipitation patterns directly determine the dryland soil hydrological pattern, which is the critical factor for maize growth in this freeze-thawing area.

### 4.2 Crop growth and N accumulation differences in plant organs

The quantification and assessment of the organ biomass in crops can help in understanding the impacts of SW and N on crop growth. This information is also necessary for assessing N leaching [37]. The crop growth is measured by the organ dry weight (Fig. 5), which indicates the crop growth rate is higher than in other drylands under warming temperatures. The yearly precipitation in 2011 was more than in 2000 (Fig. 2), but did not lead to the higher grain dry weight in 2011 (Fig. 5). The difference proves that a higher maize yield is based on a combination of climate, soil and tillage. The grain filling period in this freeze-thawing area occurs in August, at a time when temperature and precipitation are at their highest levels. The active ET facilitates the transport of water and N from soil to crop, which finally causes a higher grain filling performance [38].

The absorbed N by maize organs in the entire growth period had different allocation rates (Fig. 7), which is determined by the photosynthesis of the organs and the eco-hydrological process. The crop accumulative rate in its canopy, stem and grain varied significantly, which means the N absorption capability for tissue production of dedicated organs shifts due to the N flux from soil to above ground biomass [39]. The soil N concentration in the vertical direction proved that the subsurface has a significant variance due to the root uptake. The accumulation rates of different organs over time also follow the typical sigmoid curve [40]. The temporal patterns of N in maize organs also indicate the N absorption is not only related to the soil N concentration, but that there is a complicated systemic feedback signal within the soil-crop system [41]. The SW availability, ET and N concentration are the key signaling factors. As the soil N is transported in crops with water, there are plenty of field observations and models to identify the growth stress reaction to the SW and N conditions [42] [43]. This information provides detailed guidelines for N and water adjustment in the soil-crop system.

### 4.3 Implication for N leach control and soil N optimization

The N uptake efficiency of a crop is the main component for *NUE* and the temporal patterns in Table 4 indicated that the highest amount of grain uptake of N occurs in the graining filling and maturity periods. The maize uptake of N will increase until the optimum levels of water and N in soils are reached [44]. Overlaying this information with the leached N loading, it was found that the intensive N uptake and loss occurred at same time. The strong ET and rainfall in that time period were the reason for the N transport in the crop-soil system [45]. The soil N gap analysis revealed that the observed N gap was similar in the vertical direction, which also demonstrates that the soil N can move among the soil depths with hydrological processes and maintain the N concentration balances [3]. The temporal N analysis in crop organs, soil and leached water explain the maize growth in this freeze-thawing area, which also provides a guideline for N fertilization while, also considering SW conditions.

The N pollution to groundwater due to excessive N fertilizer application is an important environmental concern [46]. The yearly *NUE* is about 43% in this area, which is a relatively high value in comparison with similar dryland agriculture areas [47]. The N leached from farmland mainly depends on the difference between the N obtained by the soil-water-crop system and the N uptake by the crop [48]. As Table 6 shows, the leached N is mainly related to the loss of SW, which is the gap between rainfall and ET. The rainfall in August 2011 was above the long term monthly value, which caused the highest SW and N loss during the growing season. Under the normal climatic conditions, the *NUE* in this area can reach 50%. The nitrate-N is the dominant form of leached N to the groundwater, which is a direct consequence of fertilization and soil nitrification [49]. Models have been developed to optimize the fertilizer application to farmland based on a consideration of the soil hydrological process, which can help to maximize the crop yield with minimal discharge to the environment [50]. The model with local characteristic parameters, should be applied in the tillage practice to obtain an improved *NUE*.

## Conclusion

The results of this study have shown that combined conditions of soil texture, soil hydrology and precipitation were basic factors for maize growth in the freeze-thawing area in Northeast China. The rainfall significantly affected the pattern of SW content and the allocation of N in the maize organs. Precipitation was the only water source for the dryland, but the variation in SW acquisition rates and SW content remained at a stable level due to the special *albic soil* texture. The dry weight and N accumulation of maize organs in the entire growing period proved that the soil hydrological condition was also enhanced.

The N loss was dependent on both the hydrological process and the N in the water, which was then decided by the rainfall water depth and the applied N. The NO_3_-N was the dominant leachate, which was closely correlated with the amount of applied N fertilizer. The *NUE* was about 43%, which was also related to the initial soil N and maize uptake. In order to improve the *NUE* and decrease the amounts of leached nitrogen, it would be better to improve the fertilizer application amounts while giving consideration to rainfall patterns and N accumulation rates.
